# Clips and elastic band-assisted traction for biliary cannulation in a patient with an ectopic papilla within a juxtapapillary duodenal diverticulum

**DOI:** 10.1055/a-2710-6328

**Published:** 2025-10-21

**Authors:** Zhongshang Sun, Feng Pan, Shuran Hu, Rui Xie

**Affiliations:** 1 Department of Gastroenterology, The Affiliated Huaiʼan No.1 Peopleʼs Hospital of Nanjing Medical University, Huaiʼan, China; 2 Department of TCM Tui Na Therapy, Huaiʼan TCM Hospital Affiliated to Nanjing University of Chinese Medicine, Huaiʼan, China

A 63-year-old man with choledocholithiasis underwent endoscopic retrograde cholangiopancreatography (ERCP). Intraoperatively, the major duodenal papilla was located within a large juxtapapillary diverticulum. Diverticular traction rendered the papilla highly mobile, resulting in substantial difficulty with biliary cannulation. An attempt to stabilize the papilla by applying a clip to the diverticular ridge was unsuccessful ( Fig. 1 **a, b** ).

**Fig. 1:**
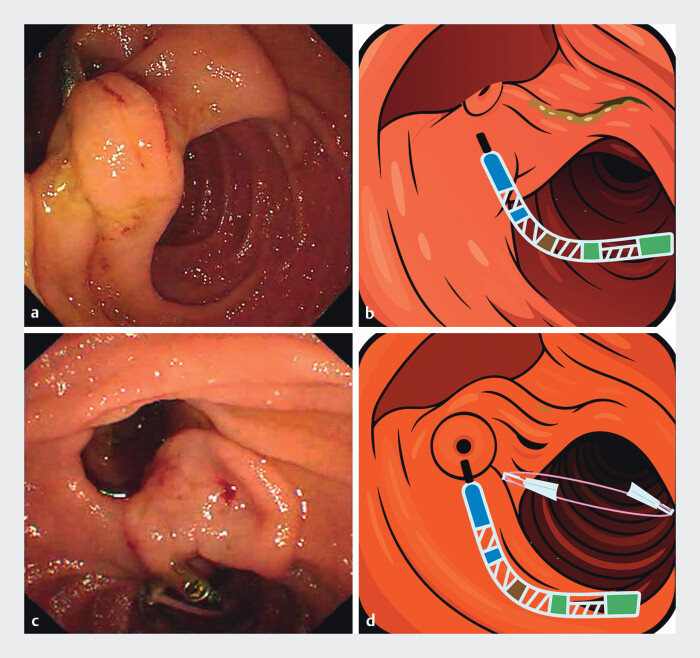
Clip–elastic band traction facilitates biliary cannulation of an intradiverticular papilla. **a** Intradiverticular major papilla with diverticular traction: papillary hypermobility leading to difficult biliary cannulation and failure of clip-only stabilization. **b** Vector-style schematic corresponding to panel **a** , illustrating papillary hypermobility due to diverticular traction. **c** Two-clip elastic-band traction stabilizes the papilla and straightens the biliary axis, enabling deep common bile duct cannulation. **d** Vector-style schematic corresponding to panel **c** , illustrating clip and band placement and papillary stabilization.

To overcome this challenge, a simple traction system was assembled using two endoscopic clips and an elastic band. The first clip was affixed to the suprapapillary duodenal wall (i.e., below the papilla) to avoid inadvertent clamping of the biliary orifice or ductal structures; the second clip was then used to anchor the elastic band to the distal duodenum, thereby optimizing papillary stabilization and exposure and straightening the biliary axis ( Fig. 1 **c, d** , Video 1 ). Following stabilization, deep cannulation of the common bile duct was achieved without difficulty. The patient tolerated the procedure well and recovered uneventfully.

Clip–elastic band traction for biliary cannulation in an intradiverticular papilla.Video 1

Cases involving an anatomic variant – specifically, an ectopic papilla located within a large duodenal diverticulum – can significantly complicate ERCP. The use of clip-assisted traction may facilitate successful cannulation in such challenging scenarios 1 . Strategic traction of peripapillary tissue using readily available endoscopic accessories can effectively stabilize the papilla and facilitate successful cannulation, offering a practical approach for managing complex biliary anatomy and potentially improving procedural outcomes.

Endoscopy_UCTN_Code_TTT_1AR_2AC

## Notes

Endoscopy E-Videos https://eref.thieme.de/e-videos
*E-Videos* is an open access online section of the journal *Endoscopy* , reporting on interesting cases and new techniques in gastroenterological endoscopy. All papers include a high-quality video and are published with a Creative Commons CC-BY license. Endoscopy E-Videos qualify for HINARI discounts and waivers and eligibility is automatically checked during the submission process. We grant 100% waivers to articles whose corresponding authors are based in Group A countries and 50% waivers to those who are based in Group B countries as classified by Research4Life (see: https://www.research4life.org/access/eligibility/ ). This section has its own submission website at https://mc.manuscriptcentral.com/e-videos .
